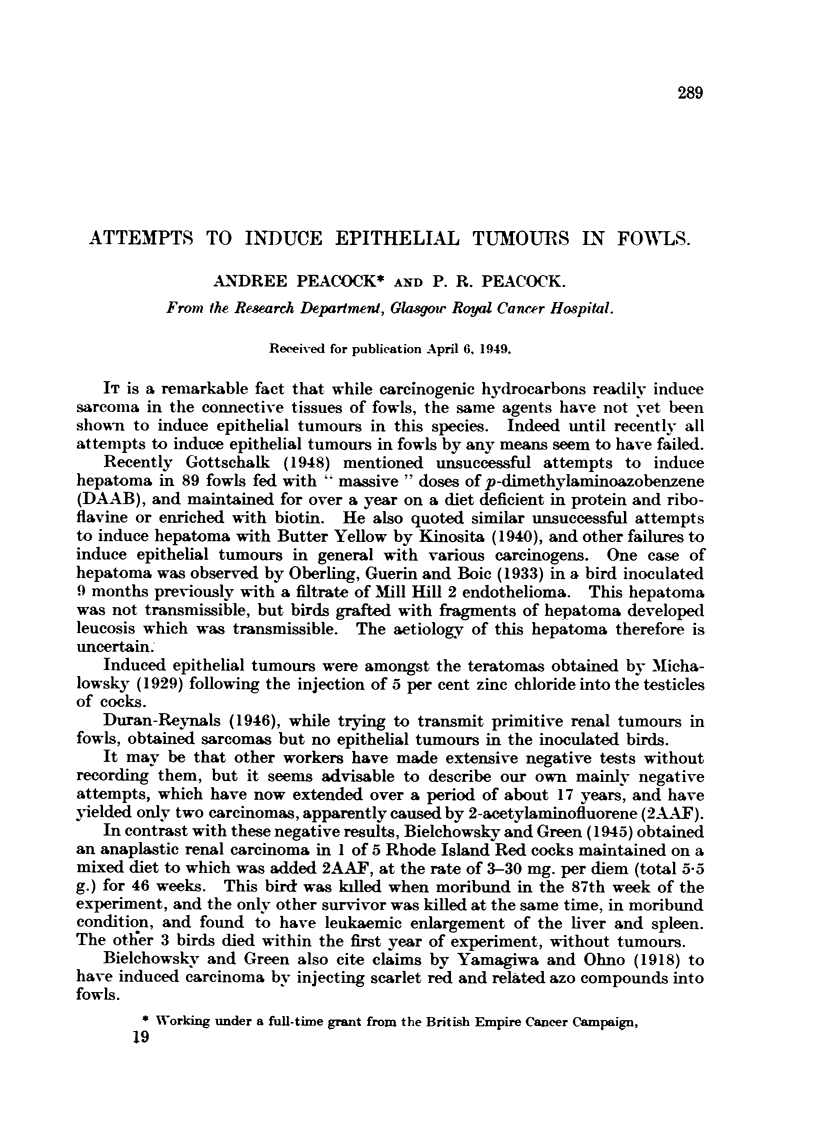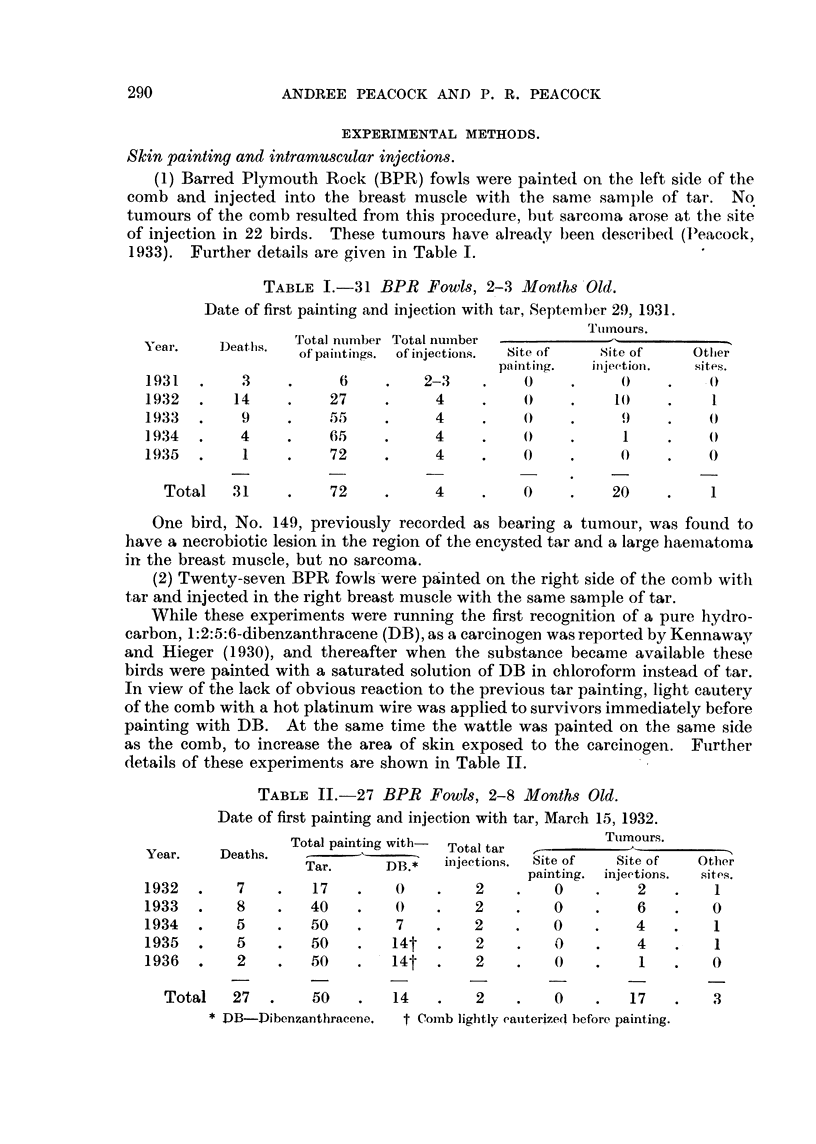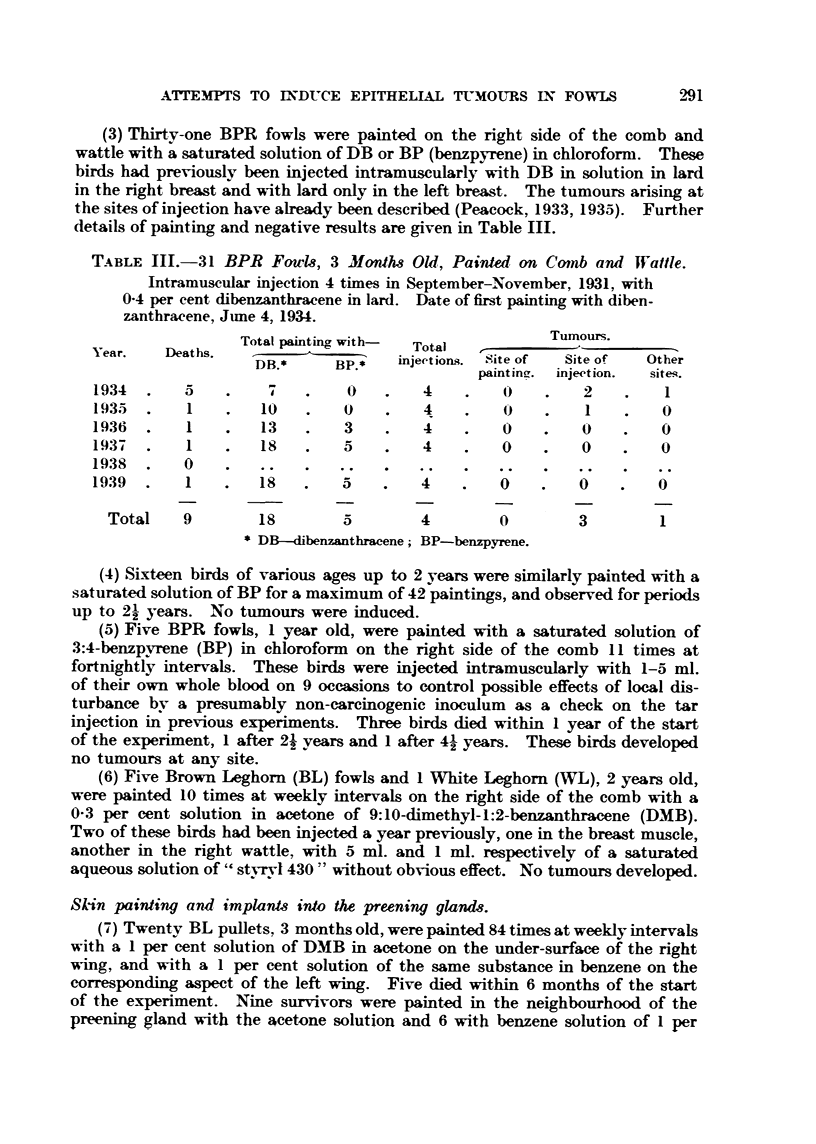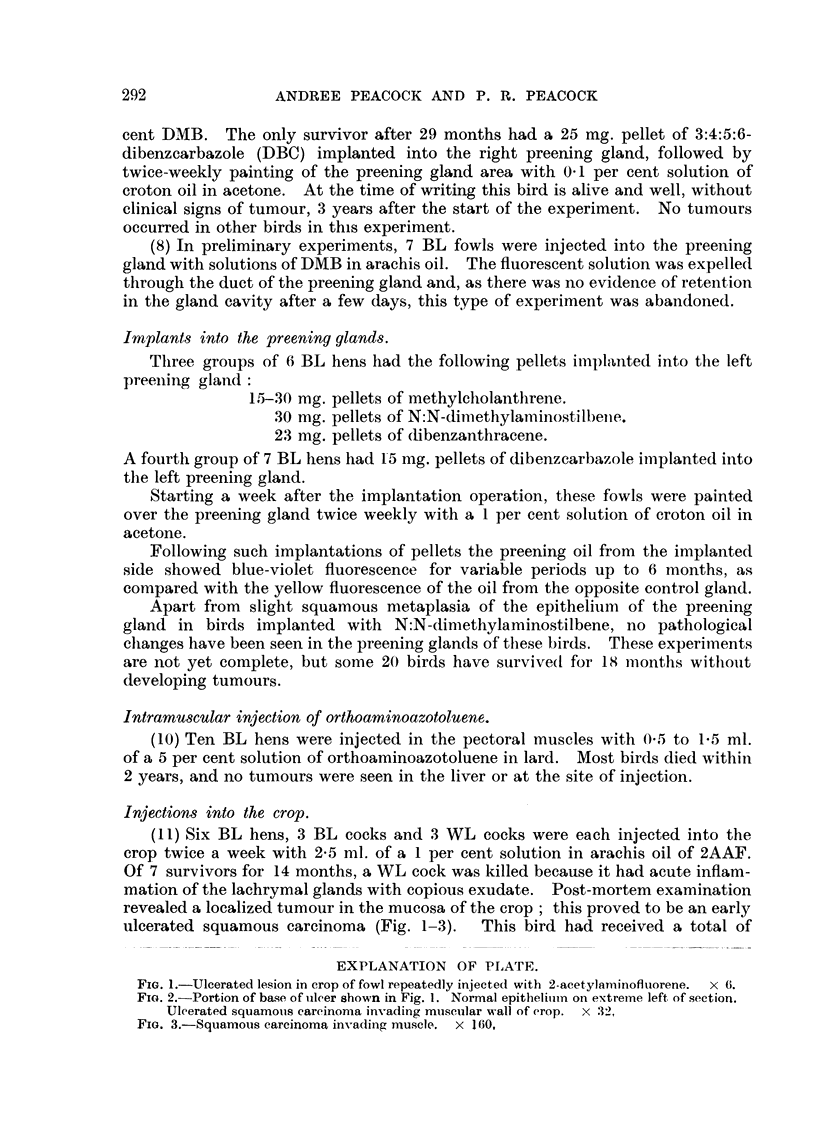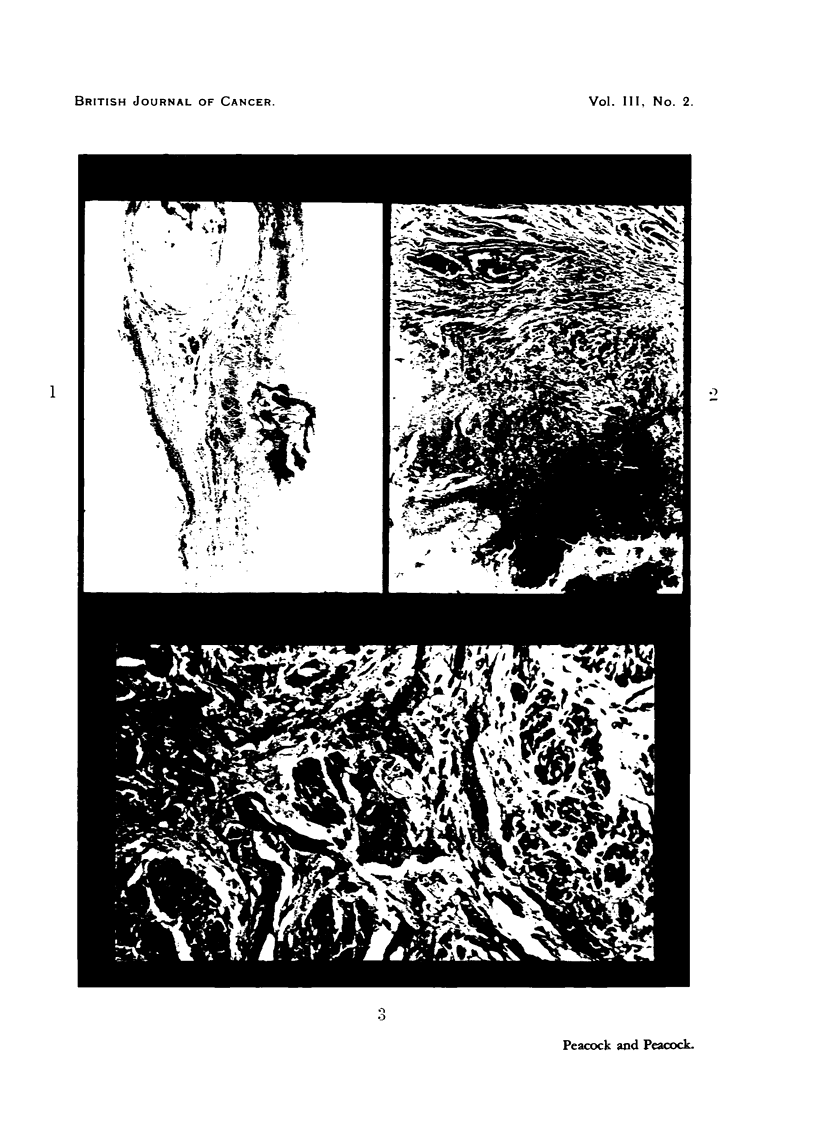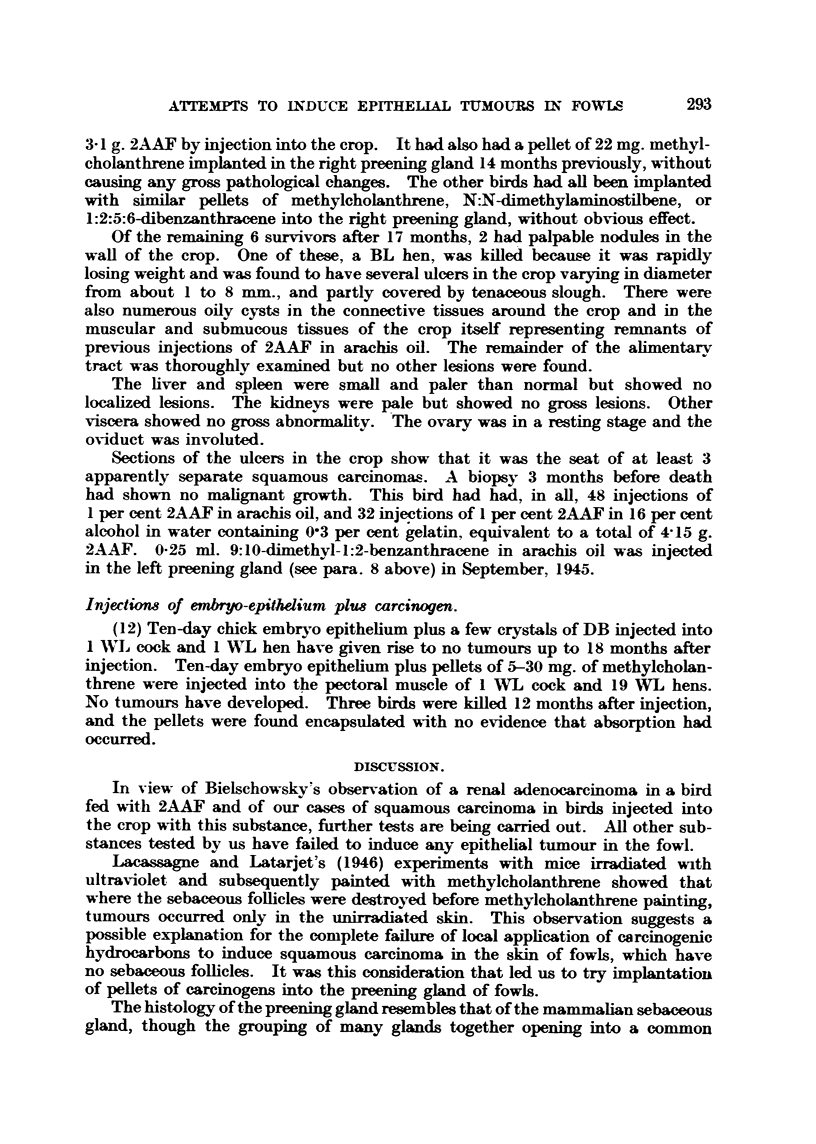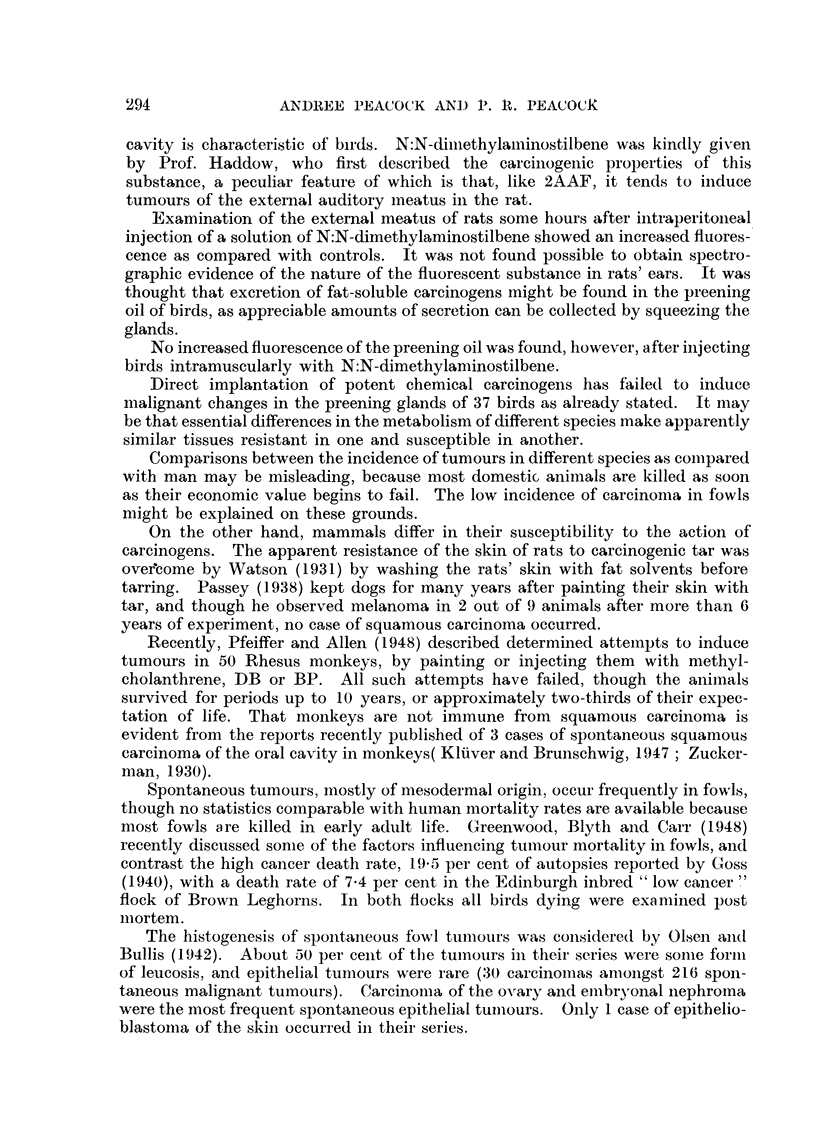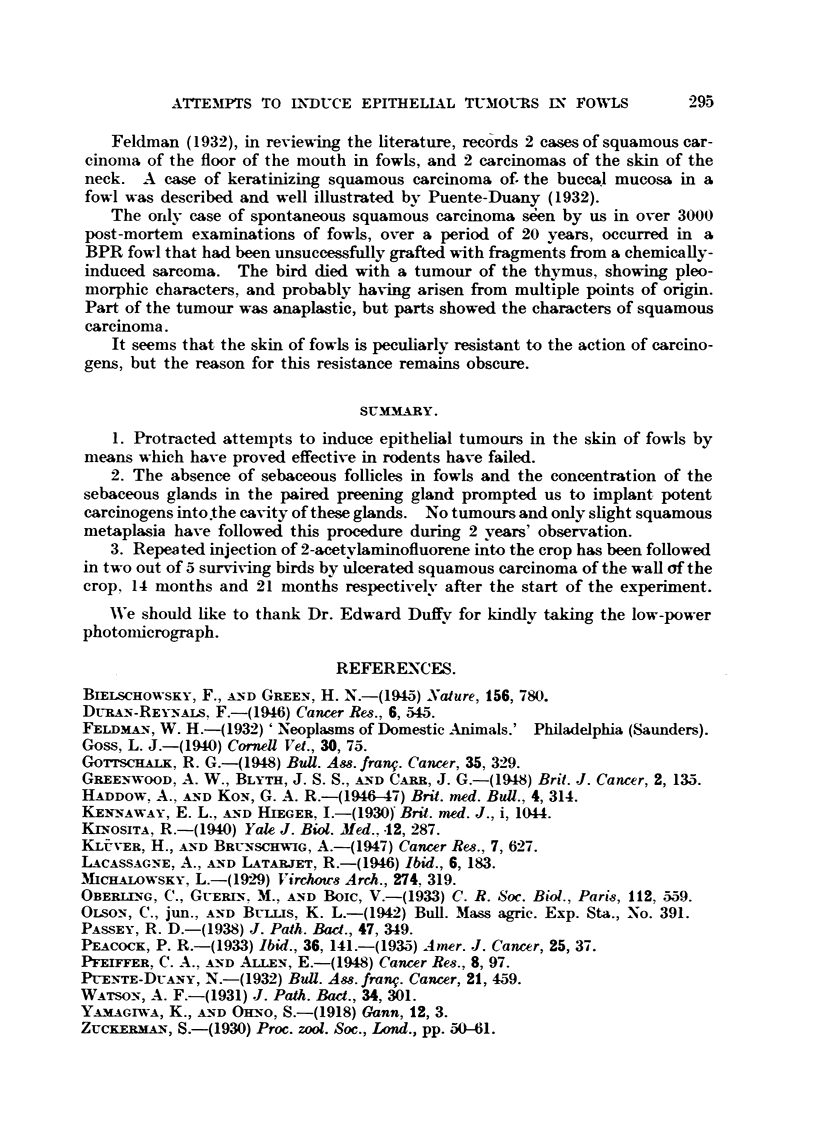# Attempts to Induce Epithelial Tumours in Fowls

**DOI:** 10.1038/bjc.1949.34

**Published:** 1949-06

**Authors:** Andree Peacock, P. R. Peacock

## Abstract

**Images:**


### ATTEMPTS TO INDUCE EPITHELIAL TUMOURS IN FOWLS.

ANDREE PEACOCK* AND P. R. PEACOCK.

*From the Research Department, Glasgow Royal Cancer Hospital.*

Received for publication April 6, 1949.

It is a remarkable fact that while carcinogenic hydrocarbons readily induce sarcoma in the connective tissues of fowls, the same agents have not yet been shown to induce epithelial tumours in this species. Indeed until recently all attempts to induce epithelial tumours in fowls by any means seem to have failed.

Recently Gottschalk (1948) mentioned unsuccessful attempts to induce hepatoma in 89 fowls fed with " massive " doses of *p*-dimethylaminoazobenzene (DAAB), and maintained for over a year on a diet deficient in protein and riboflavine or enriched with biotin. He also quoted similar unsuccessful attempts to induce hepatoma with Butter Yellow by Kinosita (1940), and other failures to induce epithelial tumours in general with various carcinogens. One case of hepatoma was observed by Oberling, Guerin and Boic (1933) in a bird inoculated 9 months previously with a filtrate of Mill Hill 2 endothelioma. This hepatoma was not transmissible, but birds grafted with fragments of hepatoma developed leucosis which was transmissible. The aetiology of this hepatoma therefore is uncertain.

Induced epithelial tumours were amongst the teratomas obtained by Michalowsky (1929) following the injection of 5 per cent zinc chloride into the testicles of cocks.

Duran-Reynals (1946), while trying to transmit primitive renal tumours in fowls, obtained sarcomas but no epithelial tumours in the inoculated birds.

It may be that other workers have made extensive negative tests without recording them, but it seems advisable to describe our own mainly negative attempts, which have now extended over a period of about 17 years, and have yielded only two carcinomas, apparently caused by 2-acetylaminofluorene (2AAF).

In contrast with these negative results, Bielchowsky and Green (1945) obtained an anaplastic renal carcinoma in 1 of 5 Rhode Island Red cocks maintained on a mixed diet to which was added 2AAF, at the rate of 3–30 mg. per diem (total 5·5 g.) for 46 weeks. This bird was killed when moribund in the 87th week of the experiment, and the only other survivor was killed at the same time, in moribund condition, and found to have leukaemic enlargement of the liver and spleen. The other 3 birds died within the first year of experiment, without tumours.

Bielchowsky and Green also cite claims by Yamagiwa and Ohno (1918) to have induced carcinoma by injecting scarlet red and related azo compounds into fowls.

* Working under a full-time grant from the British Empire Cancer Campaign,

### EXPERIMENTAL METHODS.

*Skin painting and intramuscular injections.*

(1) Barred Plymouth Rock (BPR) fowls were painted on the left side of the comb and injected into the breast muscle with the same sample of tar. No tumours of the comb resulted from this procedure, but sarcoma arose at the site of injection in 22 birds. These tumours have already been described (Peacock, 1933). Further details are given in Table I.

TABLE I.—31 *BPR Fowls*, 2–3 *Months Old.*

Date of first painting and injection with tar, September 29, 1931.

| Year. | Deaths. | Total number of paintings. | Total number of injections. | Tumours. Site of painting. | Site of injection. | Other sites. |
|---|---|---|---|---|---|---|
| 1931 | 3 | 6 | 2–3 | 0 | 0 | 0 |
| 1932 | 14 | 27 | 4 | 0 | 10 | 1 |
| 1933 | 9 | 55 | 4 | 0 | 9 | 0 |
| 1934 | 4 | 65 | 4 | 0 | 1 | 0 |
| 1935 | 1 | 72 | 4 | 0 | 0 | 0 |
| Total | 31 | 72 | 4 | 0 | 20 | 1 |

One bird, No. 149, previously recorded as bearing a tumour, was found to have a necrobiotic lesion in the region of the encysted tar and a large haematoma in the breast muscle, but no sarcoma.

(2) Twenty-seven BPR fowls were painted on the right side of the comb with tar and injected in the right breast muscle with the same sample of tar.

While these experiments were running the first recognition of a pure hydrocarbon, 1:2:5:6-dibenzanthracene (DB), as a carcinogen was reported by Kennaway and Hieger (1930), and thereafter when the substance became available these birds were painted with a saturated solution of DB in chloroform instead of tar. In view of the lack of obvious reaction to the previous tar painting, light cautery of the comb with a hot platinum wire was applied to survivors immediately before painting with DB. At the same time the wattle was painted on the same side as the comb, to increase the area of skin exposed to the carcinogen. Further details of these experiments are shown in Table II.

TABLE II.—27 *BPR Fowls*, 2–8 *Months Old.*

Date of first painting and injection with tar, March 15, 1932.

| Year. | Deaths. | Total painting with— Tar. | DB.* | Total tar injections. | Tumours. Site of painting. | Site of injections. | Other sites. |
|---|---|---|---|---|---|---|---|
| 1932 | 7 | 17 | 0 | 2 | 0 | 2 | 1 |
| 1933 | 8 | 40 | 0 | 2 | 0 | 6 | 0 |
| 1934 | 5 | 50 | 7 | 2 | 0 | 4 | 1 |
| 1935 | 5 | 50 | 14† | 2 | 0 | 4 | 1 |
| 1936 | 2 | 50 | 14† | 2 | 0 | 1 | 0 |
| Total | 27 | 50 | 14 | 2 | 0 | 17 | 3 |

* DB—Dibenzanthracene. † Comb lightly cauterized before painting.

(3) Thirty-one BPR fowls were painted on the right side of the comb and wattle with a saturated solution of DB or BP (benzpyrene) in chloroform. These birds had previously been injected intramuscularly with DB in solution in lard in the right breast and with lard only in the left breast. The tumours arising at the sites of injection have already been described (Peacock, 1933, 1935). Further details of painting and negative results are given in Table III.

TABLE III.—31 *BPR Fowls, 3 Months Old, Painted on Comb and Wattle.*
Intramuscular injection 4 times in September–November, 1931, with 0·4 per cent dibenzanthracene in lard. Date of first painting with dibenzanthracene, June 4, 1934.

| Year. | Deaths. | Total painting with— | | Total injections. | Tumours. | | |
|---|---|---|---|---|---|---|---|
| | | DB.* | BP.* | | Site of painting. | Site of injection. | Other sites. |
| 1934 | 5 | 7 | 0 | 4 | 0 | 2 | 1 |
| 1935 | 1 | 10 | 0 | 4 | 0 | 1 | 0 |
| 1936 | 1 | 13 | 3 | 4 | 0 | 0 | 0 |
| 1937 | 1 | 18 | 5 | 4 | 0 | 0 | 0 |
| 1938 | 0 | .. | .. | .. | .. | .. | .. |
| 1939 | 1 | 18 | 5 | 4 | 0 | 0 | 0 |
| Total | 9 | 18 | 5 | 4 | 0 | 3 | 1 |

* DB—dibenzanthracene ; BP—benzpyrene.

(4) Sixteen birds of various ages up to 2 years were similarly painted with a saturated solution of BP for a maximum of 42 paintings, and observed for periods up to 2½ years. No tumours were induced.

(5) Five BPR fowls, 1 year old, were painted with a saturated solution of 3:4-benzpyrene (BP) in chloroform on the right side of the comb 11 times at fortnightly intervals. These birds were injected intramuscularly with 1–5 ml. of their own whole blood on 9 occasions to control possible effects of local disturbance by a presumably non-carcinogenic inoculum as a check on the tar injection in previous experiments. Three birds died within 1 year of the start of the experiment, 1 after 2½ years and 1 after 4½ years. These birds developed no tumours at any site.

(6) Five Brown Leghorn (BL) fowls and 1 White Leghorn (WL), 2 years old, were painted 10 times at weekly intervals on the right side of the comb with a 0·3 per cent solution in acetone of 9:10-dimethyl-1:2-benzanthracene (DMB). Two of these birds had been injected a year previously, one in the breast muscle, another in the right wattle, with 5 ml. and 1 ml. respectively of a saturated aqueous solution of " styryl 430 " without obvious effect. No tumours developed.

*Skin painting and implants into the preening glands.*

(7) Twenty BL pullets, 3 months old, were painted 84 times at weekly intervals with a 1 per cent solution of DMB in acetone on the under-surface of the right wing, and with a 1 per cent solution of the same substance in benzene on the corresponding aspect of the left wing. Five died within 6 months of the start of the experiment. Nine survivors were painted in the neighbourhood of the preening gland with the acetone solution and 6 with benzene solution of 1 per cent DMB. The only survivor after 29 months had a 25 mg. pellet of 3:4:5:6-dibenzcarbazole (DBC) implanted into the right preening gland, followed by twice-weekly painting of the preening gland area with 0·1 per cent solution of croton oil in acetone. At the time of writing this bird is alive and well, without clinical signs of tumour, 3 years after the start of the experiment. No tumours occurred in other birds in this experiment.

(8) In preliminary experiments, 7 BL fowls were injected into the preening gland with solutions of DMB in arachis oil. The fluorescent solution was expelled through the duct of the preening gland and, as there was no evidence of retention in the gland cavity after a few days, this type of experiment was abandoned.

*Implants into the preening glands.*

Three groups of 6 BL hens had the following pellets implanted into the left preening gland :

15–30 mg. pellets of methylcholanthrene.
30 mg. pellets of N:N-dimethylaminostilbene.
23 mg. pellets of dibenzanthracene.

A fourth group of 7 BL hens had 15 mg. pellets of dibenzcarbazole implanted into the left preening gland.

Starting a week after the implantation operation, these fowls were painted over the preening gland twice weekly with a 1 per cent solution of croton oil in acetone.

Following such implantations of pellets the preening oil from the implanted side showed blue-violet fluorescence for variable periods up to 6 months, as compared with the yellow fluorescence of the oil from the opposite control gland.

Apart from slight squamous metaplasia of the epithelium of the preening gland in birds implanted with N:N-dimethylaminostilbene, no pathological changes have been seen in the preening glands of these birds. These experiments are not yet complete, but some 20 birds have survived for 18 months without developing tumours.

*Intramuscular injection of orthoaminoazotoluene.*

(10) Ten BL hens were injected in the pectoral muscles with 0·5 to 1·5 ml. of a 5 per cent solution of orthoaminoazotoluene in lard. Most birds died within 2 years, and no tumours were seen in the liver or at the site of injection.

*Injections into the crop.*

(11) Six BL hens, 3 BL cocks and 3 WL cocks were each injected into the crop twice a week with 2·5 ml. of a 1 per cent solution in arachis oil of 2AAF. Of 7 survivors for 14 months, a WL cock was killed because it had acute inflammation of the lachrymal glands with copious exudate. Post-mortem examination revealed a localized tumour in the mucosa of the crop ; this proved to be an early ulcerated squamous carcinoma (Fig. 1–3). This bird had received a total of

EXPLANATION OF PLATE.

FIG. 1.—Ulcerated lesion in crop of fowl repeatedly injected with 2-acetylaminofluorene. × 6.

FIG. 2.—Portion of base of ulcer shown in Fig. 1. Normal epithelium on extreme left of section. Ulcerated squamous carcinoma invading muscular wall of crop. × 32.

FIG. 3.—Squamous carcinoma invading muscle. × 160.

1 2

3

3·1 g. 2AAF by injection into the crop. It had also had a pellet of 22 mg. methylcholanthrene implanted in the right preening gland 14 months previously, without causing any gross pathological changes. The other birds had all been implanted with similar pellets of methylcholanthrene, N:N-dimethylaminostilbene, or 1:2:5:6-dibenzanthracene into the right preening gland, without obvious effect.

Of the remaining 6 survivors after 17 months, 2 had palpable nodules in the wall of the crop. One of these, a BL hen, was killed because it was rapidly losing weight and was found to have several ulcers in the crop varying in diameter from about 1 to 8 mm., and partly covered by tenaceous slough. There were also numerous oily cysts in the connective tissues around the crop and in the muscular and submucous tissues of the crop itself representing remnants of previous injections of 2AAF in arachis oil. The remainder of the alimentary tract was thoroughly examined but no other lesions were found.

The liver and spleen were small and paler than normal but showed no localized lesions. The kidneys were pale but showed no gross lesions. Other viscera showed no gross abnormality. The ovary was in a resting stage and the oviduct was involuted.

Sections of the ulcers in the crop show that it was the seat of at least 3 apparently separate squamous carcinomas. A biopsy 3 months before death had shown no malignant growth. This bird had had, in all, 48 injections of 1 per cent 2AAF in arachis oil, and 32 injections of 1 per cent 2AAF in 16 per cent alcohol in water containing 0·3 per cent gelatin, equivalent to a total of 4·15 g. 2AAF. 0·25 ml. 9:10-dimethyl-1:2-benzanthracene in arachis oil was injected in the left preening gland (see para. 8 above) in September, 1945.

*Injections of embryo-epithelium plus carcinogen.*

(12) Ten-day chick embryo epithelium plus a few crystals of DB injected into 1 WL cock and 1 WL hen have given rise to no tumours up to 18 months after injection. Ten-day embryo epithelium plus pellets of 5–30 mg. of methylcholanthrene were injected into the pectoral muscle of 1 WL cock and 19 WL hens. No tumours have developed. Three birds were killed 12 months after injection, and the pellets were found encapsulated with no evidence that absorption had occurred.

### DISCUSSION.

In view of Bielschowsky's observation of a renal adenocarcinoma in a bird fed with 2AAF and of our cases of squamous carcinoma in birds injected into the crop with this substance, further tests are being carried out. All other substances tested by us have failed to induce any epithelial tumour in the fowl.

Lacassagne and Latarjet's (1946) experiments with mice irradiated with ultraviolet and subsequently painted with methylcholanthrene showed that where the sebaceous follicles were destroyed before methylcholanthrene painting, tumours occurred only in the unirradiated skin. This observation suggests a possible explanation for the complete failure of local application of carcinogenic hydrocarbons to induce squamous carcinoma in the skin of fowls, which have no sebaceous follicles. It was this consideration that led us to try implantation of pellets of carcinogens into the preening gland of fowls.

The histology of the preening gland resembles that of the mammalian sebaceous gland, though the grouping of many glands together opening into a common cavity is characteristic of birds. N:N-dimethylaminostilbene was kindly given by Prof. Haddow, who first described the carcinogenic properties of this substance, a peculiar feature of which is that, like 2AAF, it tends to induce tumours of the external auditory meatus in the rat.

Examination of the external meatus of rats some hours after intraperitoneal injection of a solution of N:N-dimethylaminostilbene showed an increased fluorescence as compared with controls. It was not found possible to obtain spectrographic evidence of the nature of the fluorescent substance in rats' ears. It was thought that excretion of fat-soluble carcinogens might be found in the preening oil of birds, as appreciable amounts of secretion can be collected by squeezing the glands.

No increased fluorescence of the preening oil was found, however, after injecting birds intramuscularly with N:N-dimethylaminostilbene.

Direct implantation of potent chemical carcinogens has failed to induce malignant changes in the preening glands of 37 birds as already stated. It may be that essential differences in the metabolism of different species make apparently similar tissues resistant in one and susceptible in another.

Comparisons between the incidence of tumours in different species as compared with man may be misleading, because most domestic animals are killed as soon as their economic value begins to fail. The low incidence of carcinoma in fowls might be explained on these grounds.

On the other hand, mammals differ in their susceptibility to the action of carcinogens. The apparent resistance of the skin of rats to carcinogenic tar was overcome by Watson (1931) by washing the rats' skin with fat solvents before tarring. Passey (1938) kept dogs for many years after painting their skin with tar, and though he observed melanoma in 2 out of 9 animals after more than 6 years of experiment, no case of squamous carcinoma occurred.

Recently, Pfeiffer and Allen (1948) described determined attempts to induce tumours in 50 Rhesus monkeys, by painting or injecting them with methylcholanthrene, DB or BP. All such attempts have failed, though the animals survived for periods up to 10 years, or approximately two-thirds of their expectation of life. That monkeys are not immune from squamous carcinoma is evident from the reports recently published of 3 cases of spontaneous squamous carcinoma of the oral cavity in monkeys( Klüver and Brunschwig, 1947 ; Zuckerman, 1930).

Spontaneous tumours, mostly of mesodermal origin, occur frequently in fowls, though no statistics comparable with human mortality rates are available because most fowls are killed in early adult life. Greenwood, Blyth and Carr (1948) recently discussed some of the factors influencing tumour mortality in fowls, and contrast the high cancer death rate, 19·5 per cent of autopsies reported by Goss (1940), with a death rate of 7·4 per cent in the Edinburgh inbred " low cancer " flock of Brown Leghorns. In both flocks all birds dying were examined post mortem.

The histogenesis of spontaneous fowl tumours was considered by Olsen and Bullis (1942). About 50 per cent of the tumours in their series were some form of leucosis, and epithelial tumours were rare (30 carcinomas amongst 216 spontaneous malignant tumours). Carcinoma of the ovary and embryonal nephroma were the most frequent spontaneous epithelial tumours. Only 1 case of epithelioblastoma of the skin occurred in their series.

Feldman (1932), in reviewing the literature, records 2 cases of squamous carcinoma of the floor of the mouth in fowls, and 2 carcinomas of the skin of the neck. A case of keratinizing squamous carcinoma of the buccal mucosa in a fowl was described and well illustrated by Puente-Duany (1932).

The only case of spontaneous squamous carcinoma seen by us in over 3000 post-mortem examinations of fowls, over a period of 20 years, occurred in a BPR fowl that had been unsuccessfully grafted with fragments from a chemically-induced sarcoma. The bird died with a tumour of the thymus, showing pleomorphic characters, and probably having arisen from multiple points of origin. Part of the tumour was anaplastic, but parts showed the characters of squamous carcinoma.

It seems that the skin of fowls is peculiarly resistant to the action of carcinogens, but the reason for this resistance remains obscure.

### SUMMARY.

1. Protracted attempts to induce epithelial tumours in the skin of fowls by means which have proved effective in rodents have failed.

2. The absence of sebaceous follicles in fowls and the concentration of the sebaceous glands in the paired preening gland prompted us to implant potent carcinogens into the cavity of these glands. No tumours and only slight squamous metaplasia have followed this procedure during 2 years' observation.

3. Repeated injection of 2-acetylaminofluorene into the crop has been followed in two out of 5 surviving birds by ulcerated squamous carcinoma of the wall of the crop, 14 months and 21 months respectively after the start of the experiment.

We should like to thank Dr. Edward Duffy for kindly taking the low-power photomicrograph.

### REFERENCES.

Bielschowsky, F., and Green, H. N.—(1945) *Nature*, **156**, 780.

Duran-Reynals, F.—(1946) *Cancer Res.*, **6**, 545.

Feldman, W. H.—(1932) 'Neoplasms of Domestic Animals.' Philadelphia (Saunders).

Goss, L. J.—(1940) *Cornell Vet.*, **30**, 75.

Gottschalk, R. G.—(1948) *Bull. Ass. franç. Cancer*, **35**, 329.

Greenwood, A. W., Blyth, J. S. S., and Carr, J. G.—(1948) *Brit. J. Cancer*, **2**, 135.

Haddow, A., and Kon, G. A. R.—(1946–47) *Brit. med. Bull.*, **4**, 314.

Kennaway, E. L., and Hieger, I.—(1930) *Brit. med. J.*, i, 1044.

Kinosita, R.—(1940) *Yale J. Biol. Med.*, **12**, 287.

Klüver, H., and Brunschwig, A.—(1947) *Cancer Res.*, **7**, 627.

Lacassagne, A., and Latarjet, R.—(1946) *Ibid.*, **6**, 183.

Michalowsky, L.—(1929) *Virchows Arch.*, **274**, 319.

Oberling, C., Guerin, M., and Boic, V.—(1933) *C. R. Soc. Biol., Paris*, **112**, 559.

Olson, C., jun., and Bullis, K. L.—(1942) Bull. Mass agric. Exp. Sta., No. 391.

Passey, R. D.—(1938) *J. Path. Bact.*, **47**, 349.

Peacock, P. R.—(1933) *Ibid.*, **36**, 141.—(1935) *Amer. J. Cancer*, **25**, 37.

Pfeiffer, C. A., and Allen, E.—(1948) *Cancer Res.*, **8**, 97.

Puente-Duany, N.—(1932) *Bull. Ass. franç. Cancer*, **21**, 459.

Watson, A. F.—(1931) *J. Path. Bact.*, **34**, 301.

Yamagiwa, K., and Ohno, S.—(1918) *Gann*, **12**, 3.

Zuckerman, S.—(1930) *Proc. zool. Soc., Lond.*, pp. 50–61.